# Child-Friendly Neighborhood for Early Childhood: a cross-sectional study on child development and urban infrastructure

**DOI:** 10.1590/1980-220X-REEUSP-2025-0594en

**Published:** 2026-08-10

**Authors:** Claudia Nery Teixeira Palombo, Fernanda Anjos Oliveira, Ráren Paulo da Silva Araújo, Larissa Fernandes de Souza, Melissa da Silva Almeida, Clariana Vitória Ramos de Oliveira

**Affiliations:** 1 Universidade Federal da Bahia, Escola de Enfermagem, Salvador, BA, Brazil.; 2 Universidade Federal da Bahia, Faculdade de Medicina da Bahia, Salvador, BA, Brazil.; 3 Universidade de São Paulo, Escola de Enfermagem, São Paulo, SP, Brazil.; 4 University of Nevada, School of Nursing, Las Vegas, Nevada, United States of America.

**Keywords:** Child Development, Healthy City, Primary Care Nursing

## Abstract

**Objective:**

To analyze the association between the risk of developmental delay in children and mothers’ perceptions of urban infrastructure.

**Method:**

A cross-sectional study was conducted in Salvador, Bahia, with mothers and children under 6 years of age registered at health units. Child development was assessed using the Survey of Well-being of Young Children, validated for the Brazilian population, and mothers’ perceptions of urban infrastructure were assessed using the Child-Friendly Neighborhood for Early Childhood scale (green/open, playful, safe, accessible, and inclusive). Descriptive statistical analyses and logistic regression models were used. All ethical aspects were respected.

**Results:**

A total of 503 mother-child dyads participated, and the risk of developmental delay was 11%. Regarding urban infrastructure, 41% of mothers considered their neighborhood green/open, 45% playful, 37% safe, 93% accessible, and 29% inclusive. Only mothers’ perception that the neighborhood was safe was associated with a lower likelihood of risk of developmental delay in children (OR = 0.33; 95%CI: 0.15–0.71; p < 0.001).

**Conclusion:**

Maternal perception of neighborhood safety was associated with a lower likelihood of risk of developmental delay in children.

## INTRODUCTION

Estimates indicate that more than 50% of the world’s population lives in urban areas, and this percentage may reach nearly 70% by 2050^(1)^. In Brazil, the urban population accounts for 87.4% of the total population^(2)^. Compared with 2010, there was an increase of 16.6 million inhabitants in cities and a reduction of 4.3 million in the rural population^(2)^.

The country, characterized by megacities and rapidly growing medium-sized cities, faces profound social, environmental, and health inequalities^(3)^. In this context, urban infrastructure plays a decisive role in population health, especially during the first 6 years of life, a period corresponding to early childhood. During this stage, child development is marked by the acquisition of multiple cognitive, motor, and socioemotional skills, strongly influenced by the social determinants of health, which shape this process and may have lifelong repercussions^(4)^.

Safe, healthy, and accessible public spaces are essential for fostering affective bonds, ensuring emotional security, and stimulating cognitive development, highlighting the central role of territory and urban infrastructure in promoting healthy development^(5)^. Thus, urban environments that encourage free and creative play not only meet the basic needs of childhood but also strengthen community ties and the sense of belonging^(6)^.

The Legal Framework for Early Childhood (Law 13,257/2016) reinforces this perspective by establishing that states and municipalities should create public spaces focused on children’s well-being, encouraging play and creativity in a safe and inclusive manner^(7)^.

The proposal of a Child-Friendly Neighborhood for Early Childhood (CFNEC) emerges as a strategy to put these principles into practice^(8,9)^, creating urban spaces that promote the comprehensive development of young children. Driven by the Van Leer Foundation, the *Instituto de Arquitetos do Brasil*, and Urban95^(10)^, the CFNEC should consider the needs of children aged 0 to 6 years and their caregivers, structuring the urban territory based on five dimensions: being green/open, playful, safe, accessible, and inclusive^(8,9)^.

Despite the growing recognition of the importance of urban infrastructure for child development, studies on this topic have largely focused on individual or family determinants of this process^(11,12)^. International studies have advanced this perspective by showing that the territory encompasses different dimensions that affect children’s development^(5,6)^. However, in the Brazilian context, little is still known about how the physical and social characteristics of neighborhoods directly influence the development of children in early childhood, according to their caregivers’ perceptions.

Furthermore, there is no consensus regarding which urban indicators are most relevant for promoting a child-friendly environment or how these indicators manifest across different urban territories, such as the municipality of Salvador, Bahia.

Given this scenario, the present study aimed to analyze the association between the risk of developmental delay in children and urban infrastructure from mothers’ perspective. The central hypothesis was that better urban infrastructure conditions, aligned with CFNEC principles, are associated with a lower risk of developmental delay in children.

## METHOD

### Study Design

This is a cross-sectional study, part of a broader investigation aimed at understanding how territorial impact dimensions affect the health and nutritional conditions of children in early childhood in Salvador (CNPq/MCTI/FNDCT Call 18/2021 – Universal, Process 408221/2021-6). This study was guided by the Strengthening the Reporting of Observational Studies in Epidemiology^(13)^.

### Setting

The study was conducted in the municipality of Salvador, which divides its territory into 12 health districts and has 162 Basic Health Units integrated into the Primary Health Care network of the Brazilian Unified Health System^(14)^.

Located on the northeastern coast of Brazil, Salvador has an estimated population of 2,418,005 inhabitants, of whom 213,765 are children under 6 years of age^(15)^. The municipality has the fourth-largest population among Brazilian cities and is the most populous city in the Northeast region, with a population density of approximately 3,500 inhabitants/km^2(14,15)^. The Municipal Human Development Index is 0.759, reflecting important advances in areas such as health, education, and income. However, significant social inequalities persist in certain regions, characterized by high poverty rates and the presence of criminal factions linked to drug trafficking^(14)^. The health units included in this study are concentrated in these vulnerable areas.

### Population and Sample

The study population consisted of mothers and children under 6 years of age registered at municipal health units. The sample was based on data from the *Instituto Brasileiro de Geografia e Estatística* (IBGE)/2010 because, at the time the sample size was calculated, the IBGE/2022 report had not yet been released. The sample size was determined based on the proportion of children with inadequate feeding practices (p = 50%)^(16)^, according to the estimate used in the larger project. The population of children under 6 years of age registered in each health district was considered. A 95% confidence level and a 5% margin of error were adopted for sample size calculation. Based on these parameters, a sample of 388 children was required.

### Selection Criteria

Children aged 0 to 6 years accompanied by their biological mothers were included in the study. Children with chronic or neurological diseases were excluded because these conditions could affect child development assessment. For mothers with more than one child within this age range, the interview focused on the youngest child.

### Data Collection

Data were collected between January and February 2023 in 27 health units designated by the Municipal Health Department, covering the 12 health districts of Salvador. Undergraduate health students, properly trained and supervised by the project coordinator, conducted interviews with mothers in private rooms to ensure confidentiality. Each interview lasted approximately 40 minutes.

The questionnaire included questions on the family’s socioeconomic conditions and 14 questions categorized into the five CFNEC domains^(8)^: green/open (In your neighborhood, are recreational areas safely located and easily accessible? Are the streets tree-lined? Is there noise in the neighborhood? Is there garbage/sewage?); playful (Do you think your neighborhood has playful spaces or active and attractive facades for children? Are streets in your neighborhood usually closed for events/open-air markets?); safe (Do you feel safe walking or cycling in your neighborhood? Do you feel safe because your neighbors, relatives, and local shopkeepers form a reliable community network and generally engage with and look after children? Is there adequate street lighting in your neighborhood? Does your neighborhood usually experience flooding?); accessible (Does your neighborhood have adequate and well-located bus stops? Does it have adequate parking areas that do not interfere with pedestrian circulation? Is access to services such as stores, schools, daycare centers, health centers, community centers, and others easy?); and inclusive (Do the sidewalks in your neighborhood have ramps for baby strollers?).

To assess child development, the Survey of Well-being of Young Children – Brazilian version (SWYC-Br) was used, a screening instrument validated for the Brazilian population^(17,18)^. Available free of charge, the SWYC-Br assesses essential areas of child development based on caregivers’ perceptions and consists of three sections: developmental milestones; behavior and emotions; and family risk factors. In this study, only the Developmental Milestones section was applied, which includes ten questions related to children’s cognitive, motor, and language domains. The questions were directed to the mothers, without direct assessment of children, as recommended by the instrument. For each age group, specific cutoff points adapted to the Brazilian context indicate whether development is adequate or whether there is a risk of developmental delay^(17,18)^.

### Study Variables and Outcome

The predictor variables considered in this study were maternal characteristics (age and race/skin color), socioeconomic conditions (education level, employment outside the home, presence of a partner, income measured in minimum wages, and participation in income transfer programs), child characteristics (age, sex, and attendance at daycare or preschool), and CFNEC indicators according to maternal perception: green/open (presence of green areas and open spaces), playful (availability of spaces and opportunities for play), safe (perception of neighborhood safety), accessible (ease of access to services and public facilities), and inclusive (perception of social inclusion and integration). The study outcome was the risk of developmental delay.

### Data Analysis and Processing

Data were processed using Stata software, version 18.0. Initially, descriptive statistics were employed, including the calculation of frequencies, means, and standard deviations of the study variables, as well as the chi-square test, adopting a significance level of 5% to compare the risk of developmental delay across health districts. Subsequently, crude and adjusted associations between the CFNEC categories and the risk of developmental delay were examined. Logistic regression models were used for the binary versions of the outcome variables, including fixed effects for the health units.

The regression models were adjusted for sociodemographic variables to reduce bias and increase the validity of results. Maternal age, maternal education, presence of a partner, family income in minimum wages, as well as child age and sex, were included. These variables represent key sociodemographic and biological dimensions that could interfere with the association between maternal perception of the neighborhood and the risk of developmental delay. Controlling for these factors allowed the model to focus more precisely on the role of the territorial context, avoiding confounding by individual or family characteristics.

Mothers’ responses regarding CFNEC indicators were aggregated by health district and spatially represented using color gradients through a Geographic Information System platform (free QGIS software). Points identified by their geographic coordinates collected in the field were plotted on a free OpenStreetMap cartographic base (https://www.openstreetmap.org) at a scale of 1:4,500.

### Ethical Aspects

All ethical aspects were respected in accordance with Resolutions 466/2012 and 510/2016, and the project was approved by the Research Ethics Committee of the School of Nursing of *Universidade Federal da Bahia* (Certificate of Presentation for Ethical Consideration 64750722.0.0000.5531 and Approval Opinion 5.800.539).

## RESULTS

A total of 503 mother-child dyads with children under 6 years of age participated in the study. The mean maternal age was 31 years; 94% self-identified as Black or Brown; 68% had more than nine years of schooling; 64% did not work outside the home; and 66% were beneficiaries of federal government income transfer programs.

Regarding children, 51% were female, and only 54% attended daycare or preschool. The risk of developmental delay was 11% in the overall sample. Among the 67 children younger than six months, 6% presented a risk of developmental delay; among the 141 children aged between 6 months and 2 years, this percentage was 13%; and among the 295 children aged between 2 and 6 years, 11% presented a risk of developmental delay.

**Figure 1:**
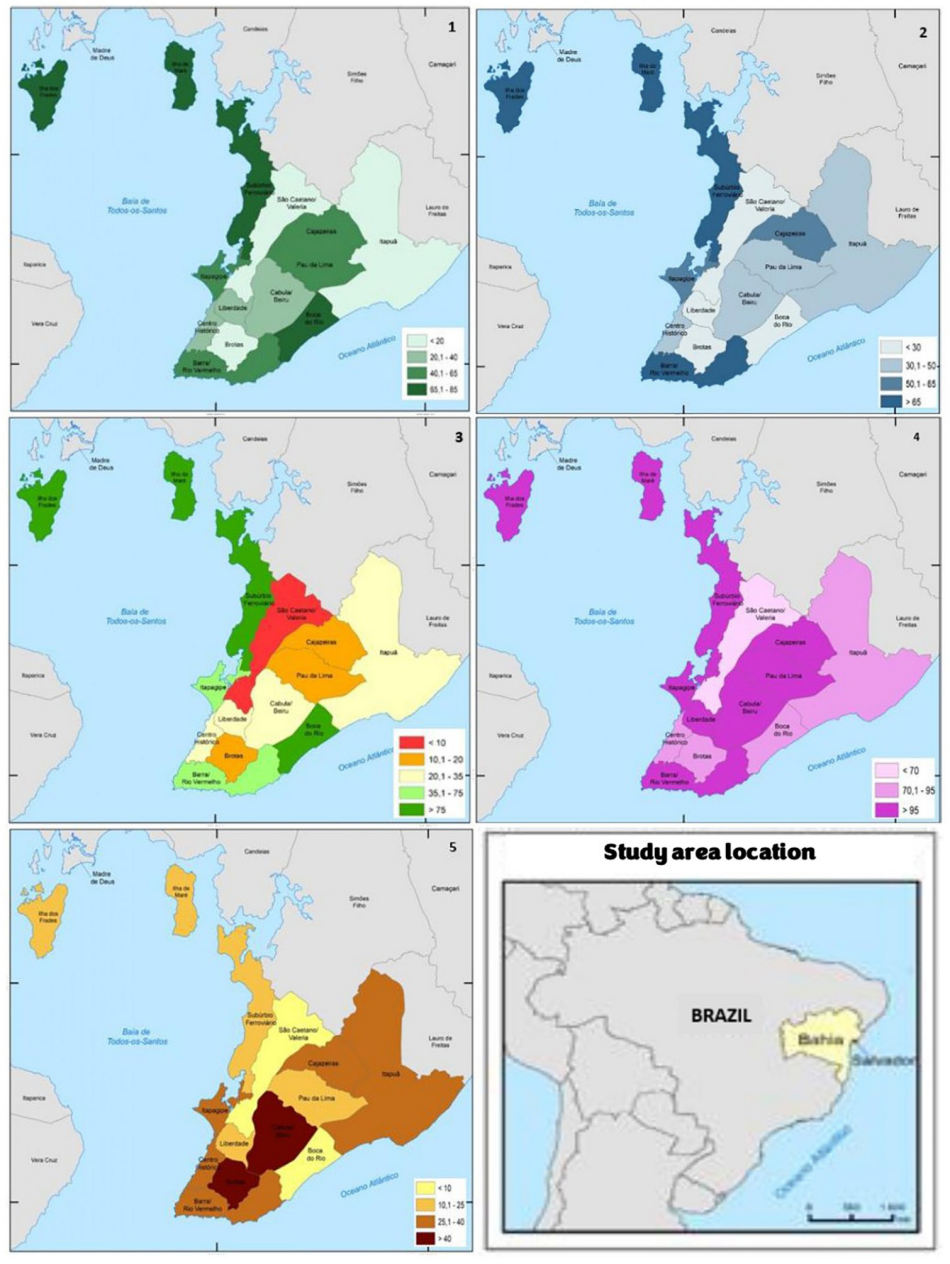
Spatial distribution of mothers’ perceptions of the Child-Friendly Neighborhood for Early Childhood indicators according to health districts (n = 503). Salvador, BA, Brazil, 2023.

Legend: Map 1 – Green/open; Map 2 – Playful; Map 3 – Safe; Map 4 – Accessible; Map 5 – Inclusive. Developed by: Luis Felipe Borges, 2024.

Figure 1 presents distribution of CFNEC indicators (green/open, playful, safe, accessible, and inclusive) across the health districts of Salvador. The *Subúrbio Ferroviário* district stood out with the highest percentages (above 80%) for virtually all indicators, according to mothers’ perceptions, except for the neighborhood being inclusive (25%). In contrast, the São Caetano/Valéria health district presented the lowest percentages for all CFNEC indicators compared with the other health districts.

Considering the five indicators for the municipality of Salvador as a whole, only 41% of mothers perceived their neighborhood as green/open; 45% considered it playful; only 37% of respondents felt their neighborhood was safe; 93% considered it accessible; and 29% perceived it as inclusive (Figure 1).

**Figure 2:**
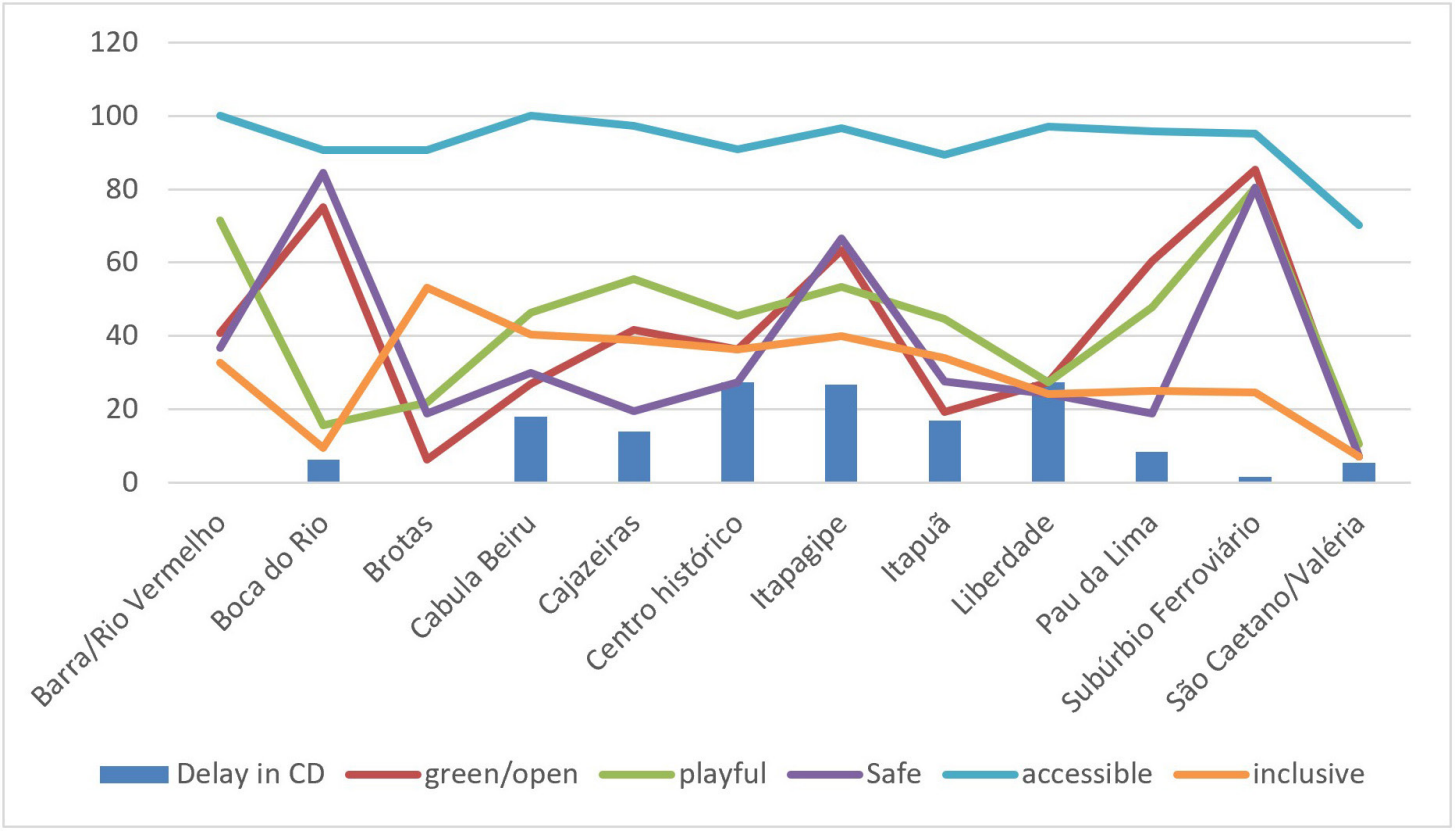
Distribution of the percentage of risk of developmental delay and mothers’ perceptions of the Child-Friendly Neighborhood for Early Childhood indicators according to health districts (n=503). Salvador, BA, Brazil, 2023.

Figure 2 presents the percentage distribution of the risk of developmental delay and the CFNEC indicators across the health districts of Salvador. The highest percentages of risk of developmental delay were observed in districts with the lowest percentages of CFNEC indicators, particularly in *Centro Histórico*, Itapuã, and Liberdade. In contrast, *Subúrbio Ferroviário* showed a low percentage of risk and better CFNEC indicators (p < 0.001).

Table 1 presents the Odds Ratio (OR), 95% Confidence Interval (95%CI), and p-values for the unadjusted and adjusted associations between the different CFNEC indicators and the risk of developmental delay, estimated using logistic regression. After adjustment for sociodemographic variables, only mothers’ perception of a “safe neighborhood” remained significantly associated with a lower likelihood of risk of developmental delay (OR = 0.33; 95%CI: 0.15–0.71; p < 0.001), suggesting a protective effect of this factor even after controlling for potential confounding variables.

**Table 1:** Associations between the risk of developmental delay and mothers’ perceptions of Child-Friendly Neighborhood for Early Childhood indicators (n = 503) – Salvador, BA, Brazil, 2023.

Indicators	Unadjusted analysis		P-value	Adjusted analysis^a^		P-value
	OR	95%CI		OR	95%CI	
Green/open	0.68	0.38; 1.24	0.22	0.79	0.43; 1.46	0.47
Playful	0.61	0.34; 1.10	0.10	0.60	0.33; 1.09	0.09
Safe	0.43	0.22; 0.85	0.01	0.33	0.15; 0.71	<0.001
Accessible	0.71	0.36; 1.36	0.30	0.93	0.30; 2.81	0.90
Inclusive	0.99	0.98; 1.09	0.59	0.85	0.49; 1.13	0.61

Legend: ^a^Model adjusted for child age and sex, maternal age and education, as well as maternal partner status and family income in minimum wages; 95%CI – 95% Confidence Interval; OR – Odds Ratio.

## DISCUSSION

The purpose of this study was to analyze the association between the risk of developmental delay and mothers’ perceptions of urban infrastructure in the municipality of Salvador, according to the CFNEC indicators (green/open, playful, safe, accessible, and inclusive). The results revealed important discrepancies between CFNEC indicators and percentages of risk of developmental delay across the different health districts. However, only mothers’ perceptions regarding the “safe neighborhood” indicator were associated with the study outcome.

Children’s everyday experiences in urban spaces during the first years of life strongly influence their development and their relationship with the environment^(4,6)^. The quality of urban infrastructure affects mothers’ well-being and, consequently, the care provided to their children^(1,19)^. This issue becomes especially relevant considering that more than 50% of the world’s population lives in urban areas^(20)^.

According to a study conducted by the *Instituto de Políticas de Transporte & Desenvolvimento* ^(21)^, caring for young children is a central factor influencing women’s mobility patterns, and territorial inequalities more intensely affect black women, women living in poverty, and female heads of household residing in peripheral areas^(21)^, characteristics similar to those of the mothers who participated in this study. This profile of social vulnerability may influence access to developmental opportunities during early childhood, reinforcing the need to address the environmental and social conditions surrounding this population.

In this study, spatial analysis of CFNEC indicators revealed inequalities among health districts that may affect the development of children under 6 years of age. While *Subúrbio Ferroviário* presented high percentages for nearly all indicators, São Caetano/Valéria stood out negatively, showing the lowest levels according to mothers’ perceptions. Across the municipality as a whole, the most critical CFNEC indicators were safety and inclusion, contrasting with the high perception of accessibility.

According to data from the Salvador Municipal Health Plan 2022–2025, the poorest health indicators are concentrated in the *Centro Histórico*, *Subúrbio Ferroviário*, and São Caetano/Valéria districts^(14)^. However, in the present study, mothers residing in *Subúrbio Ferroviário* reported a positive assessment of the CFNEC indicators. This finding may be related to public investments made in recent years, including the expansion of the primary care network (the district has the highest Family Health Team coverage in the state, with 14 teams)^(14)^; housing programs that replaced former stilt-house settlements, such as the Project *Guerreira Zeferina* ^(22)^ and *Novo* Mané Dendê^(23)^; sanitation initiatives; improvements in urban infrastructure; and implementation of the Light Rail Transit system^(24)^, which expanded and improved the transportation network.

The São Caetano/Valéria district, in turn, has been the target of intense territorial disputes involving criminal factions and conflicts with military forces^(25)^. This scenario of violence, recognized as a social determinant of health, is comparable to other Latin American contexts^(26)^, where urban violence has direct impacts on population health and mortality^(27)^.

Among the five CFNEC indicators, adjusted logistic regression showed that only mothers’ perceptions regarding the “safe neighborhood” indicator were statistically associated with a lower likelihood of risk of developmental delay, partially confirming the initial hypothesis of this study. This finding suggests that urban environments perceived as safe may exert a protective effect on early childhood development, even after controlling for sociodemographic factors.

Studies have shown that visits to parks, beaches, and green areas are associated with better overall child development^(28)^. This finding reinforces the importance of accessible natural areas in different neighborhoods and of encouraging families to take their children regularly to these spaces and promote play. This aspect is particularly relevant in Salvador, a city known for its beautiful natural landscapes. However, achieving this also requires a high-quality transportation network, in addition to ensuring that these spaces are safe. A study based on interviews with children aged 7 to 12 years living in Salvador highlighted how they perceive and appropriate the territories where they live, revealing that these spaces are not merely settings but active agents in the construction of bonds, learning experiences, and well-being^(29)^.

A safe neighborhood should allow walking, provide adequate lighting, foster trusting relationships among neighbors, and include clear demarcation and signage. These conditions enable children and caregivers to fully explore their surroundings^(10)^. However, the results of this study showed that, overall, only one-third of mothers reported feeling safe in the city.

Children exposed to neighborhoods with higher levels of social disorder are subject to toxic stress, which occurs when they experience prolonged adversity without adequate support. This type of stress can impair healthy brain development, affecting learning capacity and reasoning skills, as well as impacting other body systems, thereby increasing the risk of various diseases and growth deficits^(30,31)^. Therefore, it is essential to consider alternatives capable of mitigating the negative consequences arising from social and territorial conditions.

Urban planning guided by child development conveys a clear message that childhood is valuable and deserves care. This recognition contributes to building affective bonds between children and the territory, positively influencing how they will relate to the city throughout their lives. Every urban space is therefore potentially educational and can accommodate pedagogical intentions^(32)^.

Furthermore, providing favorable conditions for child development is more effective and less costly than attempting to reverse or mitigate the effects of early adversities. The greater the deficit produced, the more expensive it becomes to remedy later^(33)^. In the long term, children who have had fewer developmental opportunities are more likely to reproduce the intergenerational cycle of poverty^(34)^.

Studies indicate that positive experiences in early childhood, combined with high-quality interventions and services during this period, provide the foundation for the development of children’s cognitive, social, and emotional skills^(35,36)^. To achieve this, children need to be inserted into safe and healthy environments that are physically stimulating for play and exploration and where essential services are easily accessible^(5)^.

Although CFNEC indicators were assessed through mothers’ perceptions, which may be considered a limitation of this study, it is important to recognize that this perspective provides a unique and contextualized understanding of the local reality, since “no one knows a neighborhood better than its own residents”^(8)^. In addition, this study presents other limitations that help contextualize the findings and guide future research: 1) CFNEC indicators were developed based on technical and methodological guidelines but have not yet undergone a formal scientific validation process, requiring caution when interpreting the results; and 2) As this is a cross-sectional study, there are limitations regarding the ability to infer causality between urban infrastructure and the risk of developmental delay. Despite these limitations, this study advances understanding of how the urban environment may act as a protective or risk factor early in life, contributing to more effective public policies that are territorially sensitive to child development.

Regarding nurses’ practice in Primary Health Care, it is important to emphasize that the assessment of child development through screening scales or the Child Health Handbook during well-child consultations is not sufficient when used in isolation. Nurses must be familiar with the territory and advocate for the community by demanding better safety conditions, social facilities, and infrastructure. Furthermore, they should not blame families but rather help them understand the extent to which living and housing conditions interfere with children’s health and development. These responsibilities constitute essential components of nursing practice and reinforce the need for a broader and more contextualized approach.

Moreover, the findings of this study are directly aligned with the Sustainable Development Goals (SDGs), particularly SDG 11 (Sustainable Cities and Communities) and SDG 3 (Good Health and Well-being)(37). By demonstrating that safe urban conditions that favor mobility, play, and social interaction positively affect early childhood development, these findings reinforce that public policies guided by CFNEC principles represent not only a child protection agenda but also a structural strategy for building inclusive, resilient, and equitable cities. Investment in urban infrastructure sensitive to early childhood contributes to reducing territorial inequalities, promoting healthier environments, and strengthening child development trajectories with intergenerational impact, in line with the international commitments undertaken by Brazil within the framework of the 2030 Agenda^(37)^.

## CONCLUSION

This study reveals significant disparities in the quality of environments available for child development across the different health districts of the municipality of Salvador. Variations were observed in mothers’ perceptions regarding safety conditions, accessibility, green areas, inclusion, and playful environments throughout the municipality.

Although accessibility received positive assessments in all districts, investment in playful spaces and green areas was considered insufficient in many neighborhoods according to mothers’ perceptions, which may limit opportunities for healthy child development. The lack of green spaces and the low perception of inclusion in some areas highlight the urgent need for interventions focused on creating more welcoming and stimulating environments for children.

Recognizing that safety, accessibility, and the presence of playful and green spaces are interdependent and should be addressed in an integrated manner is essential for planning public policies that promote an urban environment capable of adequately supporting children’s growth and development.

Furthermore, the findings reinforce that the qualification of territories depends not only on the physical presence of urban facilities but also on how these spaces are activated and appropriated by families. In this way, more equitable urban planning that is sensitive to the needs of early childhood not only improves children’s quality of life but also contributes to the healthy social and emotional development of the entire community. In this regard, urban planning policies guided by early childhood principles should integrate infrastructure with community mobilization strategies, programming of public spaces, and strengthening of local caregiving networks, thereby expanding the reach of interventions and ensuring that the territory becomes genuinely conducive to comprehensive child development.

## DATA AVAILABILITY

In accordance with Open Science principles, the complete dataset supporting the findings of this study has been published in the Dryad Data repository, DOI: 10.5061/dryad.n2z34tnb5, available at: http://datadryad.org.
