# Supplementary material for: Child-Friendly Neighborhood for Early Childhood: a cross-sectional study on child development and urban infrastructure
Source: Rev Esc Enferm USP. 2026 Aug 10;60:e20250594. doi: 10.1590/1980-220X-REEUSP-2025-0594en (PMC13475376; doi:10.1590/1980-220X-REEUSP-2025-0594en)
Supplement: Translation

# Translation: Bairro Amigável à Primeira Infância: estudo transversal sobre desenvolvimento infantil e infraestrutura urbana

**DOI:** 10.1590/1980-220X-REEUSP-2025-0594pt

**Authors:** Claudia Nery Teixeira Palombo, Fernanda Anjos Oliveira, Ráren Paulo da Silva Araújo, Larissa Fernandes de Souza, Melissa da Silva Almeida, Clariana Vitória Ramos de Oliveira

**Affiliations:** 1 Universidade Federal da Bahia, Escola de Enfermagem, Salvador, BA, Brasil.; 2 Universidade Federal da Bahia, Faculdade de Medicina da Bahia, Salvador, BA, Brasil.; 3 Universidade de São Paulo, Escola de Enfermagem, São Paulo, SP, Brasil.; 4 University of Nevada, School of Nursing, Las Vegas, Nevada, United States of America.

**Keywords:** Desenvolvimento Infantil, Cidade Saudável, Enfermagem de Atenção Primária

## Abstract

**Objetivo:**

Analisar a associação entre o risco de atraso no desenvolvimento infantil e a percepção das mães sobre a infraestrutura urbana.

**Método:**

Estudo transversal, realizado em Salvador, Bahia, com mães e crianças menores de 6 anos cadastradas em unidades de saúde. O desenvolvimento infantil foi avaliado pelo instrumento de triagem Survey of Well-being of Young Children, validado para a população brasileira, e a percepção das mães sobre a infraestrutura urbana, pela escala Bairro Amigável à Primeira Infância (verde/livre, lúdico, seguro, acessível e inclusivo). Utilizou-se análise estatística descritiva e modelos de regressão linear. Todos os aspectos éticos foram respeitados.

**Resultados:**

Participaram 503 pares mãe-criança, e o risco de atraso no desenvolvimento foi de 11%. Quanto à infraestrutura urbana, 41% das mães consideraram o bairro verde/livre, 45%, lúdico, 37%, seguro, 93%, acessível, e 29%, inclusivo. Apenas a percepção das mães de que o bairro era seguro associou-se a uma chance menor de risco de atraso no desenvolvimento infantil (OR = 0,33; IC95%: 0,15–0,71; p < 0,001).

**Conclusão:**

A percepção materna de segurança no bairro esteve associada a uma chance menor de risco de atraso no desenvolvimento infantil.

## INTRODUÇÃO

Estimativas indicam que mais de 50% da população mundial vive em áreas urbanas, podendo esse percentual chegar a quase 70% até 2050^(1)^. No Brasil, a população urbana corresponde a 87,4% da população total^(2)^. Em comparação com 2010, houve um aumento de 16,6 milhões de habitantes nas cidades e uma redução de 4,3 milhões na população rural^(2)^.

O país, marcado por megacidades e cidades médias em rápido crescimento, enfrenta profundas desigualdades sociais, ambientais e de saúde^(3)^. Nesse contexto, a infraestrutura urbana desempenha papel decisivo na saúde da população, especialmente nos primeiros 6 anos de vida, período que corresponde à primeira infância. Nessa fase, o desenvolvimento infantil é marcado pela aquisição de múltiplas habilidades cognitivas, motoras e socioemocionais, fortemente influenciadas pelos determinantes sociais de saúde, que moldam esse processo e podem repercutir ao longo da vida^(4)^.

Espaços públicos seguros, saudáveis e acessíveis são fundamentais para favorecer vínculos afetivos, garantir a segurança emocional e estimular o desenvolvimento cognitivo, evidenciando o papel central do território e da infraestrutura urbana na promoção de um desenvolvimento saudável^(5)^. Assim, ambientes urbanos que promovem o brincar livre e criativo não apenas atendem às necessidades básicas da infância, mas também fortalecem os vínculos comunitários e o senso de pertencimento^(6)^.

O Marco Legal da Primeira Infância (Lei nº 13.257/2016) reforça essa perspectiva ao estabelecer que estados e municípios devem criar espaços públicos voltados ao bem-estar infantil, incentivando o brincar e a criatividade de maneira segura e inclusiva^(7)^.

A proposta de um Bairro Amigável à Primeira Infância (BAPI) surge como uma estratégia para concretizar esses princípios^(8,9)^, criando espaços urbanos que favoreçam o desenvolvimento integral de crianças pequenas. Impulsionado pela Fundação Van Leer, pelo Instituto de Arquitetos do Brasil e pelo *Urban95* ^(10)^, o BAPI deve considerar as necessidades de crianças de 0 a 6 anos e de seus cuidadores, estruturando o território urbano com base em cinco dimensões: ter áreas verdes e livres, ser lúdico, ser seguro, oferecer acessibilidade e ser inclusivo^(8,9)^.

Apesar do crescente reconhecimento da importância da infraestrutura urbana para o desenvolvimento infantil, os estudos relacionados à temática ainda tomam como objeto desse processo os determinantes individuais ou familiares^(11,12)^. Estudos internacionais já têm avançado nessa perspectiva, apontando que o território apresenta diferentes dimensões que afetam o desenvolvimento das crianças^(5,6)^. No entanto, no contexto brasileiro, ainda pouco se sabe sobre como as características físicas e sociais dos bairros influenciam diretamente o desenvolvimento de crianças na primeira infância, segundo a percepção de seus cuidadores.

Além disso, não há consenso sobre quais indicadores urbanos são mais relevantes para promover um ambiente favorável à infância nem sobre como esses indicadores se manifestam em diferentes territórios urbanos, como no município de Salvador, Bahia.

Diante desse cenário, o presente estudo teve como objetivo analisar a associação entre o risco de atraso no desenvolvimento infantil e a infraestrutura urbana na perspectiva das mães. A hipótese central é que melhores condições de infraestrutura urbana, alinhadas aos princípios do BAPI, estão associadas a um risco menor de atraso no desenvolvimento infantil.

## MÉTODO

### Desenho do Estudo

Trata-se de um estudo transversal, parte de uma investigação mais ampla com o objetivo de compreender como as dimensões de impacto do território afetam as condições de saúde e nutrição das crianças da primeira infância em Salvador (Edital CNPq/MCTI/FNDCT Nº 18/2021 – Universal, Processo nº 408221/2021-6). Este estudo foi orientado pela ferramenta *STrengthening the Reporting of OBservational studies in Epidemiology* ^(13)^.

### Local

O estudo foi conduzido no município de Salvador, que divide seu território em 12 distritos sanitários e conta com 162 Unidades Básicas de Saúde, integradas à rede de Atenção Primária à Saúde do Sistema Único de Saúde^(14)^.

Localizada no litoral nordestino, Salvador possui uma população estimada de 2.418.005 habitantes, dos quais 213.765 são crianças menores de 6 anos^(15)^. O município apresenta a quarta maior população entre as cidades brasileiras e é o mais populoso da região Nordeste, com densidade demográfica de aproximadamente 3,5 mil hab./km^2(14,15)^. O Índice de Desenvolvimento Humano Municipal é de 0,759, refletindo avanços importantes em áreas como saúde, educação e renda. No entanto, persistem desigualdades sociais significativas em determinadas regiões, marcadas por altos índices de pobreza e pela atuação de facções criminosas ligadas ao tráfico de drogas^(14)^. Nessas áreas vulneráveis, concentram-se as unidades de saúde incluídas neste estudo.

### População e Amostra

A população deste estudo foi composta por mães e crianças menores de 6 anos de idade cadastradas em unidades de saúde do município. A amostra considerou dados do Instituto Brasileiro de Geografia e Estatística (IBGE)/2010, pois, no período do cálculo, ainda não havia sido disponibilizado o relatório do IBGE/2022. O tamanho da amostra foi definido a partir da proporção de crianças com práticas alimentares inadequadas (p = 50%)^(16)^, conforme estimativa utilizada no projeto maior. Considerou-se a população de crianças menores de 6 anos cadastradas em cada distrito sanitário. Para o cálculo amostral, adotou-se um nível de confiança de 95% e uma margem de erro de 5%. Com base nesses parâmetros, determinou-se a necessidade de uma amostra de 388 crianças.

### Critérios de Seleção

Foram incluídas no estudo crianças de 0 a 6 anos de idade acompanhadas por suas mães biológicas. Crianças com doenças crônicas ou neurológicas foram excluídas, pois essas condições poderiam alterar a avaliação do desenvolvimento infantil. Para as mães acompanhadas por mais de um filho dentro dessa faixa etária, a entrevista foi direcionada ao filho que possuía menor idade.

### Coleta de Dados

A coleta de dados ocorreu entre janeiro e fevereiro de 2023, em 27 unidades de saúde indicadas pela Secretaria Municipal de Saúde, abrangendo os 12 distritos sanitários de Salvador. Estudantes de graduação da área da saúde, devidamente treinados e supervisionados pela coordenadora do projeto, conduziram as entrevistas com as mães em salas reservadas, garantindo privacidade. Cada entrevista teve duração aproximada de 40 minutos.

O formulário continha questões sobre as condições socioeconômicas da família e 14 questões categorizadas nos cinco eixos do BAPI^(8)^: verde e livre (No seu bairro, as áreas de lazer estão localizadas de forma segura e de fácil acesso? As ruas do seu bairro são arborizadas? Tem ruído/barulho no bairro? Lixo/esgoto?); lúdico (Você acha que seu bairro possui espaços lúdicos ou fachadas ativas e atraentes para as crianças? No seu bairro, costuma ter fechamento de ruas para eventos/feiras livres?); seguro (Você se sente segura em andar pelo seu bairro a pé ou de bicicleta? No seu bairro, você se sente segura porque seus vizinhos, parentes e comerciantes conhecidos formam uma rede comunitária confiável e geralmente se envolvem e ficam atentos à criança? Nas ruas do seu bairro, tem iluminação adequada? No seu bairro, costuma ter enchentes?); acessível (No seu bairro, tem pontos de ônibus adequados e bem localizados? No seu bairro, tem áreas de estacionamento adequadas, que não atrapalhem os pedestres? No seu bairro, é fácil o acesso a serviços, como comércio, escolas, creche, centros de saúde, centros de convivência, outros?); e inclusivo (As calçadas do seu bairro possuem rampas para carrinho de bebês?).

Para caracterizar o desenvolvimento infantil, utilizou-se o *Survey of Well-being of Young Children* (SWYC-Br), instrumento de rastreio validado para a população brasileira^(17,18)^. Disponível gratuitamente, o SWYC-Br analisa áreas essenciais do desenvolvimento infantil a partir da percepção dos cuidadores e é composto por três seções: marcos do desenvolvimento; comportamento e emoções; e fatores de risco familiares. Neste estudo, aplicou-se exclusivamente a seção “Marcos do desenvolvimento”, que reúne dez questões relativas aos domínios cognitivos, motores e de linguagem das crianças. As perguntas foram direcionadas às mães, sem avaliação direta das crianças, conforme preconizado pelo instrumento. Para cada faixa etária, pontos de corte específicos para a realidade brasileira indicam se o desenvolvimento é adequado ou se há risco de atraso^(17,18)^.

### Variáveis do Estudo e Desfecho

As variáveis preditoras consideradas neste estudo foram as características maternas (idade e raça/cor), as condições socioeconômicas (escolaridade, trabalho fora do lar, presença de parceiro, renda em salários mínimos e participação em programas de transferência de renda), as características infantis (idade, sexo e frequência à creche ou pré-escola) e os indicadores do BAPI segundo a percepção materna: verde/livre (presença de áreas verdes e espaços abertos), lúdico (disponibilidade de espaços e oportunidades para brincar), seguro (percepção de segurança no bairro), acessível (facilidade de acesso a serviços e equipamentos públicos) e inclusivo (percepção de acolhimento e integração social). O desfecho do estudo foi o risco de atraso no desenvolvimento infantil.

### Análise e Tratamento de Dados

Os dados foram processados no *software* Stata, versão 18.0. Inicialmente, empregou-se estatística descritiva, com cálculo de frequências, médias e desvios padrão das variáveis do estudo, além do teste qui-quadrado, adotando-se nível de significância de 5% para a comparação do risco de atraso no desenvolvimento entre os distritos sanitários. Em seguida, foram examinadas as associações brutas e ajustadas entre as categorias do BAPI e o risco de atraso no desenvolvimento infantil. Modelos de regressão logística foram utilizados para as versões binárias das variáveis de desfecho, incluindo efeitos fixos para as unidades de saúde.

Os modelos de regressão foram ajustados controlando variáveis sociodemográficas, com o objetivo de reduzir vieses e aumentar a validade dos resultados. Foram incluídas idade materna, escolaridade materna, presença de parceiro, renda familiar em salários mínimos, além da idade e do sexo da criança. Essas variáveis representam dimensões sociodemográficas e biológicas fundamentais que poderiam interferir na associação entre a percepção materna do bairro e o risco de atraso no desenvolvimento infantil. O controle desses fatores possibilita que o modelo se concentre de forma mais precisa no papel do contexto territorial, evitando a confusão com características individuais ou familiares.

As respostas das mães referentes aos indicadores do BAPI foram somadas por distrito sanitário e espacializadas por gradiente de cor, utilizando-se uma plataforma de Sistema de Informação Geográfica (*software* gratuito QGIS). Sobre uma base cartográfica gratuita do *Open Street Map* (https://www.openstreetmap.org) em escala de 1:4.500 foram locados os pontos identificados por suas coordenadas geográficas coletadas em campo.

### Aspectos Éticos

Todos os aspectos éticos foram respeitados, de acordo com as Resoluções n^o^ 466/2012 e n^o^ 510/2016, e o projeto foi aprovado pelo Comitê de Ética em Pesquisa da Escola de Enfermagem da Universidade Federal da Bahia (Certificado de Apresentação para Apreciação Ética nº 64750722.0.0000.5531 e Parecer nº 5.800.539).

## RESULTADOS

Participaram do estudo 503 pares de mães-crianças menores de 6 anos. A idade média das mães foi de 31 anos; 94% se autodeclararam pretas ou pardas; 68% tinham mais de nove anos de escolaridade; 64% não trabalhavam fora do lar; e 66% eram beneficiárias de programas de transferência de renda do governo federal.

Quanto às crianças, 51% eram do sexo feminino, e apenas 54% frequentavam creche ou pré-escola. O risco de atraso no desenvolvimento infantil foi de 11% na amostra total de participantes, sendo que, das 67 crianças com menos de seis meses, 6% apresentavam risco de atraso no desenvolvimento infantil; entre as 141 com idade entre 6 meses e 2 anos, esse percentual foi de 13%; e entre as 295 com idade entre 2 e 6 anos, 11% apresentavam risco de atraso no desenvolvimento infantil.

A Figura 1 apresenta a distribuição dos indicadores do BAPI (verde/live, lúdico, seguro, acessível e inclusivo) por distrito sanitário do município de Salvador. O Subúrbio Ferroviário se destaca com o maior percentual (acima de 80%) em praticamente todos os indicadores, de acordo com a percepção das mães, exceto quanto ao bairro ser inclusivo (25%). Por outro lado, o distrito sanitário de São Caetano/Valéria apresentou os menores percentuais em relação a todos os indicadores do BAPI em comparação com os demais distritos sanitários.

**Figura 1:** Distribuição espacial da percepção das mães sobre os indicadores do Bairro Amigo da Primeira Infância, de acordo com os distritos sanitários (n = 503). Salvador, BA, Brasil, 2023

Legenda: Mapa 1 – Verde/livre; Mapa 2 – Lúdico; Mapa 3 – Seguro; Mapa 4 – Acessível; Mapa 5 – Inclusivo. Elaboração: Luis Felipe Borges, 2024.

Considerando os cinco indicadores para o município de Salvador, no geral, apenas 41% das mães percebem o bairro como verde/livre; 45% o consideram lúdico; apenas 37% das entrevistadas sentem que seu bairro é seguro; 93% sentem que seu bairro é acessível; e 29% sentem que seu bairro é inclusivo (Figura 1).

A Figura 2 apresenta a distribuição percentual de risco de atraso no desenvolvimento infantil e dos indicadores do BAPI por distrito sanitário do município de Salvador. Observa-se que o os maiores percentuais de risco de atraso no desenvolvimento infantil estão relacionados aos menores percentuais dos indicadores do BAPI no Centro Histórico, em Itapuã e na Liberdade. Já no Subúrbio Ferroviário, há um baixo percentual de risco e melhores indicadores de BAPI (p < 0,001).

**Figura 2:** Distribuição do percentual de risco de atraso no desenvolvimento infantil e da percepção das mães sobre os indicadores do Bairro Amigo da Primeira Infância, de acordo com os distritos sanitários (n = 503). Salvador, BA, Brasil, 2023

A Tabela 1 apresenta a Razão de Chances (OR), o Intervalo de Confiança de 95% (IC95%) e o valor de p das associações bruta e ajustada entre os diferentes indicadores do BAPI e o risco de atraso no desenvolvimento infantil, estimadas por regressão logística. Após o ajuste para variáveis sociodemográficas, apenas a percepção das mães de “bairro seguro” permaneceu significativamente associada a menor chance de risco de atraso no desenvolvimento infantil (OR = 0,33; IC95%: 0,15–0,71; p<0,001), sugerindo um efeito protetor desse fator mesmo após o controle de potenciais fatores de confusão.

**Tabela 1:** Associações entre o risco de atraso no desenvolvimento infantil e a percepção das mães sobre os indicadores do Bairro Amigável à Primeira Infância (n = 503) – Salvador, BA, Brasil, 2023.

Indicadores	Análise bruta		Valor de p	Análise ajustada^a^		Valor de p
	OR	IC95%		OR	IC95%	
Verde/livre	0,68	0,38; 1,24	0,22	0,79	0,43; 1,46	0,47
Lúdico	0,61	0,34; 1,10	0,10	0,60	0,33; 1,09	0,09
Seguro	0,43	0,22; 0,85	0,01	0,33	0,15; 0,71	<0,001
Acessível	0,71	0,36; 1,36	0,30	0,93	0,30; 2,81	0,90
Inclusivo	0,99	0,98; 1,09	0,59	0,85	0,49; 1,13	0,61

Legenda: ^a^Modelo ajustado para idade e sexo da criança, idade e escolaridade da mãe, bem como se a mãe possuía companheiro e a renda em salários mínimos da família; 95%CI – Intervalo de Confiança de 95%; OR – *Odds Ratio*.

## DISCUSSÃO

A proposta deste estudo foi analisar a associação entre o risco de atraso no desenvolvimento infantil e a percepção das mães sobre a infraestrutura urbana do município de Salvador, segundo os indicadores do BAPI (verde/livre, lúdico, seguro, acessível e inclusivo). Os resultados apontaram discrepâncias importantes entre os indicadores do BAPI e os percentuais de risco de atraso no desenvolvimento infantil nos diferentes distritos sanitários. No entanto, somente a percepção das mães em relação ao indicador “bairro seguro” se associou ao desfecho do estudo.

As experiências do cotidiano vividas pelas crianças nos espaços urbanos nos primeiros anos de vida influenciam fortemente seu desenvolvimento e sua relação com o meio ambiente^(4,6)^. A qualidade da infraestrutura urbana afeta o bem-estar das mães e, consequentemente, o cuidado com seus filhos^(1,19)^. Esse aspecto torna-se especialmente relevante ao considerar que mais de 50% da população mundial vive em áreas urbanas^(20)^.

De acordo com estudo realizado pelo Instituto de Políticas de Transporte & Desenvolvimento^(21)^, o cuidado com os filhos pequenos é um fator central no deslocamento das mulheres, e as desigualdades territoriais afetam com mais intensidade mulheres negras, pobres e chefes de família que vivem em áreas periféricas^(21)^, similar às características das mães participantes deste estudo. Esse perfil de vulnerabilidade social pode influenciar o acesso a oportunidades de desenvolvimento na primeira infância, reforçando a necessidade de atenção às condições ambientais e sociais que cercam essa população.

Neste estudo, a análise espacial dos indicadores do BAPI mostrou desigualdades entre os distritos sanitários que podem impactar o desenvolvimento das crianças menores de 6 anos de idade. Enquanto o Subúrbio Ferroviário apresentou altos percentuais em quase todos os indicadores, São Caetano/Valéria destacou-se negativamente, com os menores índices na percepção das mães. No conjunto do município, os indicadores do BAPI mais críticos foram a segurança e a inclusão, contrastando com a alta percepção de acessibilidade.

Segundo dados do Plano Municipal de Saúde de Salvador 2022–2025, os piores indicadores de saúde estão concentrados entre os distritos Centro Histórico, Subúrbio Ferroviário e São Caetano/Valéria^(14)^. Entretanto, no presente estudo, observou-se uma avaliação positiva do BAPI por parte das mães residentes no Subúrbio Ferroviário. Esse resultado pode estar relacionado aos investimentos públicos realizados ao longo dos últimos anos, que incluem a ampliação da rede de atenção básica (o distrito tem a maior cobertura de equipes de saúde da família do estado, com 14 equipes)^(14)^; programas habitacionais que substituíram antigas palafitas, como o Projeto Guerreira Zeferina^(22)^ e o Novo Mané Dendê^(23)^; ações de saneamento; melhorias na infraestrutura urbana; e implantação do Veículo Leve sobre Trilhos^(24)^, que expandiu e qualificou a rede de transporte.

O distrito de São Caetano/Valéria, por sua vez, tem sido alvo de intensas disputas territoriais por facções criminosas e conflitos com as forças armadas^(25)^. Esse cenário de violência, reconhecido como um determinante social de saúde, é comparável a outros contextos latino-americanos^(26)^, nos quais a violência urbana exerce impactos diretos sobre a saúde e a mortalidade da população^(27)^.

Entre os cinco indicadores do BAPI, a regressão logística ajustada indicou que apenas a percepção das mães sobre o indicador “bairro seguro” apresentou associação estatisticamente significativa com uma chance menor de risco de atraso no desenvolvimento infantil, contemplando parcialmente a hipótese inicial deste estudo. Esse achado sugere que ambientes urbanos percebidos como seguros podem exercer um efeito protetor sobre o desenvolvimento na primeira infância, mesmo quando fatores sociodemográficos são controlados.

Estudos evidenciam que visitas a parques, a praias e a áreas verdes estão associadas a um melhor desenvolvimento integral infantil^(28)^. Esse achado reforça a importância da existência de áreas naturais acessíveis em diferentes bairros e do incentivo para que as famílias levem seus filhos com frequência a esses espaços e estimulem o brincar. Tal aspecto é particularmente relevante em Salvador, cidade que dispõe de belíssimas paisagens. No entanto, sabe-se que, para isso, também é necessária uma rede de transporte de qualidade, além de que esses espaços ofereçam segurança. Estudo realizado a partir de entrevistas com crianças de 7 a 12 anos de idade residentes em Salvador destacou como elas percebem e se apropriam dos territórios onde vivem, revelando que tais espaços não são apenas cenários, mas agentes ativos na construção de vínculos, aprendizagens e bem-estar^(29)^.

Um bairro seguro deve possibilitar a circulação a pé, ser bem iluminado, contar com relações de confiança entre vizinhos, além de ser demarcado e bem-sinalizado. Essas condições permitem que crianças e cuidadores explorem o entorno de forma plena^(10)^. Porém, os resultados deste estudo mostraram que, de forma geral, apenas um terço das mães relatam sentir-se seguras na cidade.

Crianças expostas a bairros com maior nível de desordem social estão sujeitas a um estresse tóxico, que ocorre quando vivenciam adversidades prolongadas sem suporte adequado. Esse tipo de estresse pode comprometer o desenvolvimento saudável do cérebro, afetando a capacidade de aprendizagem e o raciocínio, além de impactar outros sistemas do corpo, aumentando o risco de diversas doenças e de déficits no crescimento^(30,31)^. Por isso, é fundamental pensar em alternativas que possam suprir os prejuízos decorrentes das condições sociais e territoriais.

Um planejamento urbano orientado pelo desenvolvimento infantil transmite uma mensagem clara de que a infância é valiosa e merece cuidado. Essa valorização contribui para a construção de vínculos afetivos entre as crianças e o território, influenciando positivamente a forma como elas se relacionarão com a cidade ao longo da vida. Todo espaço urbano, portanto, é potencialmente educativo e pode acolher intencionalidades pedagógicas^(32)^.

Ademais, oferecer condições favoráveis ao desenvolvimento infantil é mais eficaz e menos dispendioso que tentar reverter ou mitigar os efeitos das adversidades precoces, de modo que, quanto maior o déficit produzido, mais custoso é remediá-lo posteriormente^(33)^. A longo prazo, crianças que tiveram menos oportunidades de desenvolvimento têm maior probabilidade de reproduzir o ciclo intergeracional de pobreza^(34)^.

Estudos indicam que as experiências positivas na primeira infância, aliadas a intervenções e serviços de alta qualidade nesse período, formam a base para o desenvolvimento de habilidades cognitivas, sociais e emocionais das crianças^(35,36)^. Para isso, elas precisam estar inseridas em espaços seguros e saudáveis, fisicamente estimulantes para brincar, explorar e onde serviços essenciais sejam de fácil acesso^(5)^.

Apesar de os indicadores do BAPI terem sido avaliados por meio da percepção das mães, o que pode ser considerado uma limitação deste estudo, é importante reconhecer que essa perspectiva oferece uma compreensão única e contextualizada da realidade local, já que “ninguém conhece melhor um bairro que seus próprios moradores”^(8)^. Além disso, este estudo apresenta outras limitações que contribuem para contextualizar os achados e orientar futuras pesquisas: 1) Os indicadores do BAPI foram desenvolvidos com base em diretrizes técnicas e metodológicas, mas ainda não passaram por um processo formal de validação científica, o que exige cautela na interpretação dos resultados; e 2) Por se tratar de um estudo transversal, há limitações na capacidade de inferir causalidade entre a infraestrutura urbana e os riscos de atraso no desenvolvimento infantil. Apesar disso, este estudo avança na compreensão de como o ambiente urbano pode atuar como fator de proteção ou risco no início da vida, contribuindo para políticas públicas mais eficazes e territorialmente sensíveis ao desenvolvimento infantil.

Já no âmbito da atuação do enfermeiro na Atenção Primária à Saúde, é importante ressaltar que a avaliação do desenvolvimento infantil, por meio de escalas ou da Caderneta da Criança, em consultas de puericultura, não se mostra suficiente de forma isolada. O enfermeiro deve conhecer o território e atuar em defesa da comunidade, requisitando melhores condições de segurança, equipamentos sociais e infraestrutura. Além disso, não deve culpabilizar as famílias, mas sim ajudá-las a compreender o quanto as condições de vida e moradia interferem na saúde e no desenvolvimento infantil. Tais atribuições configuram responsabilidades essenciais do enfermeiro, reforçando a necessidade de uma abordagem ampliada e contextualizada.

Além disso, os achados deste estudo se alinham diretamente aos Objetivos de Desenvolvimento Sustentável (ODS), especialmente ao ODS 11 (Cidades e Comunidades Sustentáveis) e ao ODS 3 (Saúde e Bem-estar)^(37)^. Ao evidenciar que condições urbanas seguras e favoráveis à circulação, ao brincar e ao convívio impactam positivamente o desenvolvimento na primeira infância, reforça-se que políticas públicas orientadas pelos princípios do BAPI representam não apenas uma agenda de proteção à infância, mas também uma estratégia estruturante para a construção de cidades inclusivas, resilientes e equitativas. O investimento em infraestrutura urbana sensível à primeira infância contribui para reduzir desigualdades territoriais, promover ambientes mais saudáveis e fortalecer trajetórias de desenvolvimento infantil com impacto intergeracional, em consonância com os compromissos internacionais assumidos pelo Brasil no âmbito da Agenda 2030^(37)^.

## CONCLUSÃO

Este estudo revela disparidades significativas na qualidade dos ambientes oferecidos para o desenvolvimento infantil nos diferentes distritos sanitários do município de Salvador. Observa-se que existe uma variação na percepção das mães quanto às condições de segurança, à acessibilidade, às áreas verdes, à inclusão e aos ambientes lúdicos no município.

Embora a acessibilidade tenha recebido avaliações positivas em todos os distritos, o investimento em espaços lúdicos e áreas verdes foi insuficiente em muitos bairros, de acordo com a percepção das mães, o que pode limitar as oportunidades de desenvolvimento saudável das crianças. A falta de espaços verdes e a baixa percepção de inclusão em algumas áreas destacam a necessidade urgente de intervenções focadas na criação de ambientes mais acolhedores e estimulantes para as crianças.

Reconhecer que a segurança, a acessibilidade e a presença de espaços lúdicos e verdes são interdependentes e devem ser abordados de forma integrada para promover um ambiente urbano que suporte adequadamente o crescimento e o desenvolvimento das crianças é fundamental para o planejamento de políticas públicas.

Ademais, os achados reforçam que a qualificação dos territórios não depende apenas da presença física dos equipamentos urbanos, mas também da forma como esses espaços são ativados e apropriados pelas famílias. Dessa maneira, um planejamento urbano mais equitativo e sensível às necessidades da primeira infância não apenas melhora a qualidade de vida das crianças, mas também contribui para o desenvolvimento social e emocional saudável de toda a comunidade. Nesse sentido, políticas de urbanismo orientadas pela primeira infância devem articular infraestrutura com estratégias de mobilização comunitária, programação dos espaços públicos e fortalecimento das redes locais de cuidado, ampliando o alcance das intervenções e garantindo que o território se torne verdadeiramente propício ao desenvolvimento integral.

## DISPONIBILIDADE DE DADOS

Conforme preconizado pela Ciência Aberta, todo o conjunto de dados que dá suporte aos resultados deste estudo foi publicado no repositório *Dryad Data*, DOI: 10.5061/dryad.n2z34tnb5, disponível em: http://datadryad.org.

# Translation: Barrio Amigable para la Infancia: un estudio transversal sobre el desarrollo infantil y la infraestructura urbana

**DOI:** 10.1590/1980-220X-REEUSP-2025-0594es

**Authors:** Claudia Nery Teixeira Palombo, Fernanda Anjos Oliveira, Ráren Paulo da Silva Araújo, Larissa Fernandes de Souza, Melissa da Silva Almeida, Clariana Vitória Ramos de Oliveira

**Affiliations:** 1 Universidade Federal da Bahia, Escola de Enfermagem, Salvador, BA, Brasil.; 2 Universidade Federal da Bahia, Faculdade de Medicina da Bahia, Salvador, BA, Brasil.; 3 Universidade de São Paulo, Escola de Enfermagem, São Paulo, SP, Brasil.; 4 University of Nevada, School of Nursing, Las Vegas, Nevada, United States of America.

**Keywords:** Desarrollo Infantil, Ciudad Saludable, Enfermería de Atención Primaria

## Abstract

**Objetivo:**

Analizar la asociación entre el riesgo de retraso en el desarrollo infantil y la percepción materna de la infraestructura urbana.

**Método:**

Se realizó un estudio transversal en Salvador, Bahía, con madres y niños menores de 6 años registrados en centros de salud. El desarrollo infantil se evaluó mediante el Survey of Well-being of Young Children, validado para la población brasileña, y la percepción materna de la infraestructura urbana se evaluó mediante la escala de Vecindario Amigo de la Infancia (verde/libre, lúdico, seguro, accesible e inclusivo). Se utilizaron análisis estadísticos descriptivos y modelos de regresión lineal. Se respetaron todos los aspectos éticos.

**Resultados:**

Participaron 503 parejas madre-hijo, y el riesgo de retraso del desarrollo fue del 11%. En cuanto a la infraestructura urbana, el 41% de las madres consideró el vecindario verde/libre, el 45% lúdico, el 37% seguro, el 93% accesible y el 29% inclusivo. Solo la percepción de las madres sobre la seguridad del vecindario se asoció con una menor probabilidad de riesgo de retraso del desarrollo (OR = 0,33; IC95%: 0,15–0,71; p < 0,001).

**Conclusión:**

La percepción materna de la seguridad del vecindario se asoció con una menor probabilidad de riesgo de retraso del desarrollo.

## INTRODUCCIÓN

Las estimaciones indican que más del 50 % de la población mundial vive en áreas urbanas, y este porcentaje podría llegar a casi el 70 % para 2050^(1)^. En Brasil, la población urbana representa el 87,4 % de la población total^(2)^. En comparación con 2010, hubo un aumento de 16,6 millones de habitantes en las ciudades y una reducción de 4,3 millones en la población rural^(2)^.

El país, caracterizado por megaciudades y ciudades medianas en rápido crecimiento, enfrenta profundas desigualdades sociales, ambientales y de salud^(3)^. En este contexto, la infraestructura urbana desempeña un papel decisivo en la salud de la población, especialmente en los primeros 6 años de vida, período que corresponde a la primera infancia. En esta etapa, el desarrollo infantil se caracteriza por la adquisición de múltiples habilidades cognitivas, motoras y socioemocionales, fuertemente influenciadas por los determinantes sociales de la salud, que moldean este proceso y pueden tener repercusiones a lo largo de toda la vida^(4)^.

Los espacios públicos seguros, saludables y accesibles son fundamentales para favorecer los vínculos afectivos, garantizar la seguridad emocional y estimular el desarrollo cognitivo, lo que pone de manifiesto el papel central del territorio y de la infraestructura urbana en la promoción de un desarrollo saludable^(5)^. Así, los entornos urbanos que promueven el juego libre y creativo no solo satisfacen las necesidades básicas de la infancia, sino que también fortalecen los vínculos comunitarios y el sentido de pertenencia^(6)^.

El Marco Legal de la Primera Infancia (Ley n.º 13.257/2016) refuerza esta perspectiva al establecer que los estados y municipios deben crear espacios públicos orientados al bienestar infantil, fomentando el juego y la creatividad de manera segura e inclusiva^(7)^.

La propuesta de un Barrio Amigo de la Primera Infancia (BAPI) surge como una estrategia para concretar estos principios^(8,9)^, creando espacios urbanos que favorezcan el desarrollo integral de los niños pequeños. Impulsado por la Fundación Van Leer, el Instituto de Arquitectos de Brasil y *Urban95* ^(10)^, el BAPI debe considerar las necesidades de los niños de 0 a 6 años y de sus cuidadores, estructurando el territorio urbano en base a cinco dimensiones: contar con áreas verdes y libres, ser lúdico, ser seguro, ofrecer accesibilidad y ser inclusivo^(8,9)^.

A pesar del creciente reconocimiento de la importancia de la infraestructura urbana para el desarrollo infantil, los estudios relacionados con el tema aún toman como objeto de este proceso los determinantes individuales o familiares^(11,12)^. Estudios internacionales ya han avanzado en esta perspectiva, señalando que el territorio presenta diferentes dimensiones que afectan el desarrollo de los niños^(5,6)^. Sin embargo, en el contexto brasileño, aún se sabe poco sobre cómo las características físicas y sociales de los barrios influyen directamente en el desarrollo de los niños en la primera infancia, según la percepción de sus cuidadores.

Además, no hay consenso sobre qué indicadores urbanos son más relevantes para promover un entorno favorable a la infancia ni sobre cómo se manifiestan estos indicadores en diferentes territorios urbanos, como en el municipio de Salvador, Bahía.

Ante este escenario, el presente estudio tuvo como objetivo analizar la asociación entre el riesgo de retraso en el desarrollo infantil y la infraestructura urbana desde la perspectiva de las madres. La hipótesis central es que unas mejores condiciones de infraestructura urbana, alineadas con los principios del BAPI, están asociadas a un menor riesgo de retraso en el desarrollo infantil.

## MÉTODO

### Diseño del Estudio

Se trata de un estudio transversal, que forma parte de una investigación más amplia con el objetivo de comprender cómo las dimensiones de impacto del territorio afectan las condiciones de salud y nutrición de los niños en la primera infancia en Salvador (Convocatoria CNPq/MCTI/FNDCT N.º 18/2021 – Universal, Expediente N.º 408221/2021-6). Este estudio se orientó por la herramienta *STrengthening the Reporting of OBservational studies in Epidemiology* ^(13)^.

### Lugar

El estudio se llevó a cabo en el municipio de Salvador, que divide su territorio en 12 distritos sanitarios y cuenta con 162 Unidades Básicas de Salud, integradas a la red de Atención Primaria de Salud del Sistema Único de Salud^(14)^.

Situada en la costa nordeste, Salvador tiene una población estimada de 2 418 005 habitantes, de los cuales 213 765 son niños menores de 6 años^(15)^. El municipio presenta la cuarta población más grande entre las ciudades brasileñas y es la más poblada de la región noreste, con una densidad demográfica de aproximadamente 3500 hab./km^2(14,15)^. El Índice de Desarrollo Humano Municipal es de 0,759, lo que refleja avances importantes en áreas como la salud, la educación y los ingresos. Sin embargo, persisten desigualdades sociales significativas en determinadas regiones, marcadas por altos índices de pobreza y por la actuación de facciones criminales vinculadas al tráfico de drogas^(14)^. En estas áreas vulnerables se concentran las unidades de salud incluidas en este estudio.

### Población y Muestra

La población de este estudio estuvo compuesta por madres y niños menores de 6 años registrados en unidades de salud del municipio. La muestra consideró datos del Instituto Brasileño de Geografía y Estadística (IBGE)/2010, ya que, en el período del cálculo, aún no se había publicado el informe del IBGE/2022. El tamaño de la muestra se definió a partir de la proporción de niños con prácticas alimentarias inadecuadas (p = 50 %)^(16)^, según la estimación utilizada en el proyecto más amplio. Se consideró la población de niños menores de 6 años registrados en cada distrito sanitario. Para el cálculo de la muestra, se adoptó un nivel de confianza del 95 % y un margen de error del 5 %. Basándose en estos parámetros, se determinó la necesidad de una muestra de 388 niños.

### Criterios de Selección

Se incluyeron en el estudio niños de 0 a 6 años de edad acompañados por sus madres biológicas. Se excluyó a los niños con enfermedades crónicas o neurológicas, ya que estas afecciones podrían alterar la evaluación del desarrollo infantil. En el caso de las madres acompañadas por más de un hijo dentro de ese rango de edad, la entrevista se centró en el hijo de menor edad.

### Recolección de Datos

La recolección de datos se llevó a cabo entre enero y febrero de 2023, en 27 centros de salud indicados por la Secretaría Municipal de Salud, abarcando los 12 distritos sanitarios de Salvador. Estudiantes de pregrado del área de la salud, debidamente capacitados y supervisados por la coordinadora del proyecto, realizaron las entrevistas con las madres en salas reservadas, garantizando la privacidad.Cada entrevista duró aproximadamente 40 minutos.

El formulario contenía preguntas sobre las condiciones socioeconómicas de la familia y 14 preguntas categorizadas en los cinco ejes del BAPI^(8)^: verde y libre (¿En su barrio, las áreas de esparcimiento están ubicadas de manera segura y de fácil acceso? ¿Las calles de su barrio tienen árboles? ¿Hay ruido en el barrio? ¿Basura/alcantarillado?); lúdico (¿Cree que su barrio cuenta con espacios lúdicos o fachadas activas y atractivas para los niños? ¿En su barrio se suelen cerrar calles para eventos/ferias al aire libre?); seguro (¿Se siente segura al caminar por su barrio a pie o en bicicleta? ¿En su barrio se siente segura porque sus vecinos, familiares y comerciantes conocidos forman una red comunitaria confiable y, por lo general, se involucran y están atentos a los niños? ¿Hay iluminación adecuada en las calles de su barrio? ¿Su barrio suele sufrir inundaciones?); accesible (¿Hay paradas de autobús adecuadas y bien ubicadas en su barrio? ¿Hay en su barrio áreas de estacionamiento adecuadas que no obstaculicen el paso de los peatones? ¿Es fácil en su barrio acceder a servicios como comercios, escuelas, guarderías, centros de salud, centros comunitarios y otros?); e inclusivo (¿Tienen las aceras de su barrio rampas para carritos?).

Para caracterizar el desarrollo infantil, se utilizó el *Survey of Well-being of Young Children* (SWYC-Br), un instrumento de detección validado para la población brasileña^(17,18)^. Disponible de forma gratuita, el SWYC-Br analiza áreas esenciales del desarrollo infantil a partir de la percepción de los cuidadores y se compone de tres secciones: hitos del desarrollo; comportamiento y emociones; y factores de riesgo familiares. En este estudio, se aplicó exclusivamente la sección “Hitos del desarrollo”, que reúne diez preguntas relacionadas con los ámbitos cognitivo, motor y del lenguaje de los niños. Las preguntas se dirigieron a las madres, sin evaluación directa de los niños, tal como lo recomienda el instrumento. Para cada grupo de edad, los puntos de corte específicos para la realidad brasileña indican si el desarrollo es adecuado o si existe riesgo de retraso^(17,18)^.

### Variables del Estudio y Resultado

Las variables predictivas consideradas en este estudio fueron las características maternas (edad y raza/color), las condiciones socioeconómicas (nivel educativo, trabajo fuera del hogar, presencia de pareja, ingresos en salarios mínimos y participación en programas de transferencia de ingresos), las características infantiles (edad, sexo y asistencia a la guardería o preescolar) y los indicadores del BAPI según la percepción materna: verde/libre (presencia de áreas verdes y espacios abiertos), lúdico (disponibilidad de espacios y oportunidades para jugar), seguro (percepción de seguridad en el barrio), accesible (facilidad de acceso a servicios e instalaciones públicas) e inclusivo (percepción de acogida e integración social). El resultado del estudio fue el riesgo de retraso en el desarrollo infantil.

### Análisis y Procesamiento de Datos

Los datos se procesaron en el *software* Stata, versión 18.0. Inicialmente, se empleó estadística descriptiva, con cálculo de frecuencias, medias y desviaciones estándar de las variables del estudio, además de la prueba de Chi-cuadrado, adoptando un nivel de significación del 5 % para la comparación del riesgo de retraso en el desarrollo entre los distritos sanitarios.

A continuación, se examinaron las asociaciones brutas y ajustadas entre las categorías del BAPI y el riesgo de retraso en el desarrollo infantil. Se utilizaron modelos de regresión logística para las versiones binarias de las variables de resultado, incluyendo efectos fijos para las unidades de salud.

Los modelos de regresión se ajustaron controlando variables sociodemográficas, con el objetivo de reducir sesgos y aumentar la validez de los resultados. Se incluyeron la edad materna, el nivel educativo de la madre, la presencia de una pareja y el ingreso familiar en salarios mínimos, además de la edad y el sexo del niño. Estas variables representan dimensiones sociodemográficas y biológicas fundamentales que podrían interferir en la asociación entre la percepción materna del barrio y el riesgo de retraso en el desarrollo infantil. El control de estos factores permite que el modelo se concentre de manera más precisa en el papel del contexto territorial, evitando la confusión con características individuales o familiares.

Las respuestas de las madres referentes a los indicadores del BAPI se sumaron por distrito sanitario y se espacializaron mediante un gradiente de color, utilizando una plataforma de Sistema de Información Geográfica (*software* gratuito QGIS). Sobre una base cartográfica gratuita de *Open Street Map* (https://www.openstreetmap.org) a escala 1:4.500 se ubicaron los puntos identificados por sus coordenadas geográficas recolectadas en el campo.

### Aspectos Éticos

Se respetaron todos los aspectos éticos, de acuerdo con las Resoluciones n^º^ 466/2012 y n.^º^ 510/2016, y el proyecto fue aprobado por el Comité de Ética en Investigación de la Escuela de Enfermería de la Universidad Federal de Bahía (Certificado de Presentación para Apreciación Ética n.º 64750722.0.0000.5531 y Dictamen n.º 5.800.539).

## RESULTADOS

Participaron en el estudio 503 pares de madres e hijos menores de 6 años. La edad media de las madres fue de 31 años; el 94 % se autodefinieron como negras o mestizas; el 68 % tenían más de nueve años de escolaridad; el 64 % no trabajaban fuera del hogar; y el 66 % eran beneficiarias de programas de transferencia de ingresos del gobierno federal.

En cuanto a los niños, el 51 % eran niñas, y solo el 54 % asistía a guardería o preescolar. El riesgo de retraso en el desarrollo infantil fue del 11 % en la muestra total de participantes; de los 67 niños menores de seis meses, el 6 % presentaba riesgo de retraso en el desarrollo infantil; entre las 141 con edades comprendidas entre los 6 meses y los 2 años, este porcentaje fue del 13 %; y entre las 295 con edades comprendidas entre los 2 y los 6 años, el 11 % presentaba riesgo de retraso en el desarrollo infantil.

La Figura 1 presenta la distribución de los indicadores del BAPI (verde/vivo, lúdico, seguro, accesible e inclusivo) por distrito sanitario del municipio de Salvador. El Subúrbio Ferroviário destaca con el mayor porcentaje (por encima del 80 %) en prácticamente todos los indicadores, según la percepción de las madres, excepto en cuanto a que el barrio sea inclusivo (25 %). Por otro lado, el distrito sanitario de São Caetano/Valéria presentó los porcentajes más bajos en relación con todos los indicadores del BAPI en comparación con los demás distritos sanitarios.

**Figura 1:** Distribución espacial de la percepción de las madres sobre los indicadores del “Barrio Amigo de la Primera Infancia”, según los distritos sanitarios (n = 503). Salvador, BA, Brasil, 2023.

Leyenda: Mapa 1 – Verde/libre; Mapa 2 – Lúdico; Mapa 3 – Seguro; Mapa 4 – Accesible; Mapa 5 – Inclusivo. Elaboración: Luis Felipe Borges, 2024.

Teniendo en cuenta los cinco indicadores para el municipio de Salvador, en general, solo el 41 % de las madres perciben el barrio como verde/libre; el 45 % lo consideran lúdico; solo el 37 % de las entrevistadas sienten que su barrio es seguro; el 93 % sienten que su barrio es accesible; y el 29 % sienten que su barrio es inclusivo (Figura 1).

La Figura 2 presenta la distribución porcentual del riesgo de retraso en el desarrollo infantil y de los indicadores del BAPI por distrito sanitario del municipio de Salvador. Se observa que los mayores porcentajes de riesgo de retraso en el desarrollo infantil están relacionados con los menores porcentajes de los indicadores del BAPI en el Centro Histórico, en Itapuã y en Liberdade. En cambio, en el Subúrbio Ferroviário, hay un bajo porcentaje de riesgo y mejores indicadores del BAPI (p < 0,001).

**Figura 2:** Distribución del porcentaje de riesgo de retraso en el desarrollo infantil y de la percepción de las madres sobre los indicadores del programa “Barrio Amigo de la Primera Infancia”, según los distritos sanitarios (n = 503). Salvador, BA, Brasil, 2023.

La Tabla 1 presenta la razón de posibilidades (OR), el intervalo de confianza del 95 % (IC95 %) y el valor p de las asociaciones brutas y ajustadas entre los diferentes indicadores del BAPI y el riesgo de retraso en el desarrollo infantil, estimadas mediante regresión logística. Tras el ajuste por variables sociodemográficas, solo la percepción de las madres de un “barrio seguro” siguió estando significativamente asociada con una menor probabilidad de riesgo de retraso en el desarrollo infantil (OR = 0,33; IC95%: 0,15–0,71; p<0,001), lo que sugiere un efecto protector de este factor incluso tras el control de posibles factores de confusión.

**Tabla 1:** Asociaciones entre el riesgo de retraso en el desarrollo infantil y la percepción de las madres sobre los indicadores del Barrio Amigable de la Primera Infancia (n = 503) – Salvador, BA, Brasil, 2023.

Indicadores	Análisis bruta		Valor de p	Análisis ajustada^a^		Valor de p
	OR	IC95%		OR	IC95%	
Verde/libre	0,68	0,38; 1,24	0,22	0,79	0,43; 1,46	0,47
Lúdico	0,61	0,34; 1,10	0,10	0,60	0,33; 1,09	0,09
Seguro	0,43	0,22; 0,85	0,01	0,33	0,15; 0,71	<0,001
Accesible	0,71	0,36; 1,36	0,30	0,93	0,30; 2,81	0,90
Inclusivo	0,99	0,98; 1,09	0,59	0,85	0,49; 1,13	0,61

Leyenda: ^a^Modelo ajustado por la edad y el sexo del niño, la edad y el nivel educativo de la madre, así como por si la madre tenía pareja y los ingresos familiares en salarios mínimos; IC del 95 %: Intervalo de confianza del 95 %; OR: *Odds Ratio*.

## DISCUSIÓN

El objetivo de este estudio fue analizar la asociación entre el riesgo de retraso en el desarrollo infantil y la percepción de las madres sobre la infraestructura urbana del municipio de Salvador, según los indicadores del BAPI (verde/libre, lúdico, seguro, accesible e inclusivo). Los resultados señalaron discrepancias importantes entre los indicadores del BAPI y los porcentajes de riesgo de retraso en el desarrollo infantil en los diferentes distritos sanitarios. Sin embargo, solo la percepción de las madres en relación con el indicador “barrio seguro” se asoció con el resultado del estudio.

Las experiencias cotidianas que viven los niños en los espacios urbanos durante los primeros años de vida influyen fuertemente en su desarrollo y su relación con el entorno^(4,6)^. La calidad de la infraestructura urbana afecta el bienestar de las madres y, en consecuencia, el cuidado de sus hijos^(1,19)^. Este aspecto cobra especial relevancia si se considera que más del 50 % de la población mundial vive en áreas urbanas^(20)^.

Según un estudio realizado por el Instituto de Políticas de Transporte y Desarrollo^(21)^, el cuidado de los hijos pequeños es un factor central en los desplazamientos de las mujeres, y las desigualdades territoriales afectan con mayor intensidad a las mujeres negras, pobres y jefas de familia que viven en áreas periféricas^(21)^, características similares a las de las madres participantes en este estudio. Este perfil de vulnerabilidad social puede influir en el acceso a oportunidades de desarrollo en la primera infancia, lo que refuerza la necesidad de prestar atención a las condiciones ambientales y sociales que rodean a esta población.

En este estudio, el análisis espacial de los indicadores del BAPI mostró desigualdades entre los distritos sanitarios que pueden afectar el desarrollo de los niños menores de 6 años. Mientras que el Subúrbio Ferroviário presentó altos porcentajes en casi todos los indicadores, São Caetano/Valéria se destacó negativamente, con los índices más bajos en la percepción de las madres. En el conjunto del municipio, los indicadores del BAPI más críticos fueron la seguridad y la inclusión, en contraste con la alta percepción de accesibilidad.

Según datos del Plan Municipal de Salud de Salvador 2022–2025, los peores indicadores de salud se concentran en los distritos Centro Histórico, Subúrbio Ferroviário y São Caetano/Valéria^(14)^. Sin embargo, en el presente estudio se observó una evaluación positiva del BAPI por parte de las madres residentes en el Subúrbio Ferroviário. Este resultado puede estar relacionado con las inversiones públicas realizadas a lo largo de los últimos años, que incluyen la ampliación de la red de atención primaria (el distrito cuenta con la mayor cobertura de equipos de salud familiar del estado, con 14 equipos)^(14)^; programas de vivienda que sustituyeron antiguas casas sobre pilotes, como el Proyecto Guerreira Zeferina^(22)^ y el Novo Mané Dendê^(23)^; acciones de saneamiento; mejoras en la infraestructura urbana; y la implementación del Vehículo Ligero sobre Riel^(24)^, que amplió y mejoró la red de transporte.

El distrito de São Caetano/Valéria, por su parte, ha sido objeto de intensas disputas territoriales entre facciones criminales y conflictos con las fuerzas armadas^(25)^. Este escenario de violencia, reconocido como un determinante social de la salud, es comparable a otros contextos latinoamericanos^(26)^, en los que la violencia urbana ejerce impactos directos sobre la salud y la mortalidad de la población^(27)^.

Entre los cinco indicadores del BAPI, la regresión logística ajustada indicó que solo la percepción de las madres sobre el indicador “barrio seguro” presentó una asociación estadísticamente significativa con una menor probabilidad de riesgo de retraso en el desarrollo infantil, lo que confirma parcialmente la hipótesis inicial de este estudio. Este hallazgo sugiere que los entornos urbanos percibidos como seguros pueden ejercer un efecto protector sobre el desarrollo en la primera infancia, incluso cuando se controlan los factores sociodemográficos.

Los estudios evidencian que las visitas a parques, playas y áreas verdes están asociadas a un mejor desarrollo integral infantil^(28)^. Este hallazgo refuerza la importancia de la existencia de áreas naturales accesibles en diferentes barrios y de incentivar a las familias para que lleven a sus hijos con frecuencia a estos espacios y estimulen el juego. Este aspecto es particularmente relevante en Salvador, una ciudad que cuenta con paisajes hermosísimos. Sin embargo, se sabe que, para ello, también es necesaria una red de transporte de calidad, además de que estos espacios ofrezcan seguridad. Un estudio realizado a partir de entrevistas con niños de 7 a 12 años residentes en Salvador destacó cómo perciben y se apropian de los territorios donde viven, revelando que dichos espacios no son solo escenarios, sino agentes activos en la construcción de vínculos, aprendizajes y bienestar^(29)^.

Un barrio seguro debe permitir la circulación a pie, estar bien iluminado, contar con relaciones de confianza entre vecinos, además de estar delimitado y bien señalizado. Estas condiciones permiten que los niños y sus cuidadores exploren el entorno plenamente^(10)^. Sin embargo, los resultados de este estudio mostraron que, en general, solo un tercio de las madres reportan sentirse seguras en la ciudad.

Los niños expuestos a barrios con un mayor nivel de desorden social están sujetos a un estrés tóxico, que se produce cuando experimentan adversidades prolongadas sin el apoyo adecuado. Este tipo de estrés puede comprometer el desarrollo saludable del cerebro, afectando la capacidad de aprendizaje y el razonamiento, además de impactar otros sistemas del cuerpo, aumentando el riesgo de diversas enfermedades y de déficits en el crecimiento^(30,31)^. Por ello, es fundamental pensar en alternativas que puedan suplir los perjuicios derivados de las condiciones sociales y territoriales.

Una planificación urbana orientada al desarrollo infantil transmite un mensaje claro de que la infancia es valiosa y merece cuidados. Esta valoración contribuye a la construcción de vínculos afectivos entre los niños y el territorio, influyendo positivamente en la forma en que se relacionarán con la ciudad a lo largo de su vida. Todo espacio urbano, por lo tanto, es potencialmente educativo y puede acoger intencionalidades pedagógicas^(32)^.

Además, ofrecer condiciones favorables para el desarrollo infantil es más eficaz y menos costoso que intentar revertir o mitigar los efectos de las adversidades tempranas, de modo que, cuanto mayor es el déficit producido, más costoso resulta remediarlo posteriormente^(33)^. A largo plazo, los niños que han tenido menos oportunidades de desarrollo tienen mayor probabilidad de reproducir el ciclo intergeneracional de la pobreza^(34)^.

Los estudios indican que las experiencias positivas en la primera infancia, junto con intervenciones y servicios de alta calidad en ese período, constituyen la base para el desarrollo de las habilidades cognitivas, sociales y emocionales de los niños^(35,36)^. Para ello, deben estar insertados en espacios seguros y saludables, físicamente estimulantes para jugar y explorar, y donde los servicios esenciales sean de fácil acceso^(5)^.

Aunque los indicadores del BAPI se evaluaron a través de la percepción de las madres, lo que puede considerarse una limitación de este estudio, es importante reconocer que esta perspectiva ofrece una comprensión única y contextualizada de la realidad local, ya que “nadie conoce mejor un barrio que sus propios residentes”^(8)^. Además, este estudio presenta otras limitaciones que contribuyen a contextualizar los hallazgos y orientar futuras investigaciones: 1) Los indicadores del BAPI se desarrollaron con base en directrices técnicas y metodológicas, pero aún no han pasado por un proceso formal de validación científica, lo que exige cautela en la interpretación de los resultados; y 2) Al tratarse de un estudio transversal, existen limitaciones en la capacidad de inferir causalidad entre la infraestructura urbana y los riesgos de retraso en el desarrollo infantil. A pesar de ello, este estudio avanza en la comprensión de cómo el entorno urbano puede actuar como factor de protección o de riesgo en los primeros años de vida, contribuyendo a políticas públicas más eficaces y territorialmente sensibles al desarrollo infantil.

En cuanto al ámbito de actuación del enfermero en la Atención Primaria de Salud, es importante destacar que la evaluación del desarrollo infantil, mediante escalas o la Cartilla de Salud del Niño, en las consultas de puericultura, no resulta suficiente de manera aislada. El enfermero debe conocer el territorio y actuar en defensa de la comunidad, exigiendo mejores condiciones de seguridad, equipamientos sociales e infraestructura. Además, no debe culpar a las familias, sino ayudarlas a comprender en qué medida las condiciones de vida y de vivienda interfieren en la salud y el desarrollo infantil. Tales atribuciones constituyen responsabilidades esenciales del enfermero, lo que refuerza la necesidad de un enfoque ampliado y contextualizado.

Además, los hallazgos de este estudio se alinean directamente con los Objetivos de Desarrollo Sostenible (ODS), especialmente con el ODS 11 (Ciudades y comunidades sostenibles) y el ODS 3 (Salud y bienestar)^(37)^. Al evidenciar que las condiciones urbanas seguras y favorables para la circulación, el juego y la convivencia tienen un impacto positivo en el desarrollo de la primera infancia, se refuerza la idea de que las políticas públicas orientadas por los principios del BAPI representan no solo una agenda de protección de la infancia, sino también una estrategia estructurante para la construcción de ciudades inclusivas, resilientes y equitativas. La inversión en infraestructura urbana sensible a la primera infancia contribuye a reducir las desigualdades territoriales, promover entornos más saludables y fortalecer las trayectorias de desarrollo infantil con impacto intergeneracional, en consonancia con los compromisos internacionales asumidos por Brasil en el marco de la Agenda 2030^(37)^.

## CONCLUSIÓN

Este estudio revela disparidades significativas en la calidad de los entornos ofrecidos para el desarrollo infantil en los diferentes distritos sanitarios del municipio de Salvador. Se observa que existe una variación en la percepción de las madres respecto a las condiciones de seguridad, la accesibilidad, las áreas verdes, la inclusión y los entornos lúdicos en el municipio.

Aunque la accesibilidad recibió valoraciones positivas en todos los distritos, la inversión en espacios lúdicos y áreas verdes fue insuficiente en muchos barrios, según la percepción de las madres, lo que puede limitar las oportunidades de desarrollo saludable de los niños.

La falta de espacios verdes y la baja percepción de inclusión en algunas áreas resaltan la necesidad urgente de intervenciones enfocadas en la creación de entornos más acogedores y estimulantes para los niños.

Reconocer que la seguridad, la accesibilidad y la presencia de espacios lúdicos y verdes son interdependientes y deben abordarse de manera integrada para promover un entorno urbano que respalde adecuadamente el crecimiento y el desarrollo de los niños es fundamental para la planificación de políticas públicas.

Además, los hallazgos refuerzan que la calidad de los territorios no depende solo de la presencia física de los equipamientos urbanos, sino también de la forma en que las familias activan y se apropian de esos espacios. De esta manera, una planificación urbana más equitativa y sensible a las necesidades de la primera infancia no solo mejora la calidad de vida de los niños, sino que también contribuye al desarrollo social y emocional saludable de toda la comunidad. En este sentido, las políticas de urbanismo orientadas a la primera infancia deben articular la infraestructura con estrategias de movilización comunitaria, la programación de los espacios públicos y el fortalecimiento de las redes locales de cuidado, ampliando el alcance de las intervenciones y garantizando que el territorio se convierta verdaderamente en un entorno propicio para el desarrollo integral.

## DISPONIBILIDAD DE DATOS

De acuerdo con los principios de la Ciencia Abierta, todo el conjunto de datos que respalda los resultados de este estudio se ha publicado en el repositorio *Dryad Data*, DOI: 10.5061/dryad.n2z34tnb5, disponible en: http://datadryad.org.
